# Gender Barriers to Immunization: A Synthesis of UNICEF’s Analyses to Advance Equity and Coverage

**DOI:** 10.3390/vaccines13101059

**Published:** 2025-10-16

**Authors:** Cristián Mansilla, Alinane Kamlongera, Ibrahim Dadari

**Affiliations:** 1 Gender Equality Section, Programme Group, UNICEF, New York, NY 10017, USA; camansil@gmail.com; 2 Immunization Section, Programme Group, UNICEF, New York, NY 10017, USA; idadari@unicef.org

**Keywords:** gender analysis, health inequalities, immunization, Vaccine Inequity

## Abstract

Background/objectives: Despite global efforts to improve childhood immunization rates, gender-related barriers continue to hinder equitable access to vaccines worldwide. This study synthesizes gender barrier analyses conducted in various countries to better understand these challenges. This evidence synthesis aims to (1) identify the main gender-related barriers affecting immunization focusing on zero-dose targets, HPV, and COVID-19 vaccination campaigns; and (2) summarize key recommendations and lessons that have emerged from countries to overcome those gender barriers. Methods: A documentary analysis was used by reviewing data from gender barrier analyses that were conducted by multiple governments with UNICEF support. The study classified barriers using the socio-ecological model (SEM), encompassing systemic, health service, community, household, and individual-level gender barriers. Descriptive statistics and inductive thematic coding were used to analyze data. Results: This synthesis includes 24 documents representing gender barrier analyses across 29 countries. Findings highlight multiple barriers, including systemic discrimination against women in public and healthcare spaces, limited political will to address gender disparities, and limited (sex)-disaggregated and gender data. At the community and household levels, social norms restrict women’s autonomy in seeking immunization services, while household duties (culturally assigned to women) also restrict their access to immunization services. Adolescents face additional challenges, particularly regarding HPV vaccination, due to misconceptions and stigma from families and peers. Conclusions: Addressing gender-related barriers requires a multi-level approach, integrating gender-responsive policies, and comprehensively addressing gender barriers that are hindering the progress of vaccination efforts. UNICEF’s commitment to gender-responsive immunization strategies is critical for achieving the Immunization Agenda 2030 and ensuring equitable vaccine access for all.

## 1. Introduction

More than 154 million lives are estimated to have been saved over the last 50 years through the power of vaccination, with 95% of these being children under 5 years [1]. Despite this progress, there remain substantial inequities with vaccination coverage stagnating over the past decade, contributed in part by the impact of the COVID-19 pandemic [2]. The COVID-19 pandemic disrupted livelihoods and health systems, but also enabled an unprecedented vaccination effort, where multiple learnings were curated to help design better strategies to improve vaccination uptake [3]. While substantial progress has been made and the pandemic is over, there are still important gaps—some of which emanate from the pre-pandemic period—in reducing the number of zero-dose children and achieving greater childhood vaccination levels, which is especially relevant for achieving the Global Immunization Agenda 2030 (IA2030) and the Sustainable Development Goals (SDGs) [4,5].

The IA2030 comes at a critical time, outlining a modern vision of a world in which vaccination should be universally available, leaving no one behind [6,7]. It further highlights the critical importance of recognizing and overcoming both direct and indirect barriers to immunization access, as well as the importance of considering barriers shaped by the gender of caregivers and health workers [8]. Strategic priorities (SP) 3 and 4 highlight the importance of extending immunization services to everyone regardless of location, age, gender, or life-course stage [9]. This agenda advocates for a greater involvement of women in decision-making at all levels, as well as promoting a better understanding of the influence of gender on vaccination access.

Gender barriers to immunization refer to the social, cultural, economic, and institutional obstacles rooted in gender norms and inequalities that hinder individuals—particularly women and caregivers—from accessing and utilizing immunization services for themselves or their children. These barriers go beyond biological sex differences and encompass a range of gendered factors that influence health-seeking behaviour, decision-making, and access to health services [10,11,12].

Multiple barriers that hinder the achievement of better results in childhood immunization have been mapped. Whereas common barriers can be found at the parents/caretakers, providers, and health-system level, evidence suggests that gender barriers are an important factor to consider when designing efforts to increase childhood immunization [6]. Hence, limited decision-making power over resources and institutional inequalities create reduced women’s access to vaccination, which directly affects children’s access to vaccination services [13]. Similarly, gendered harassment presents an obstacle to the work of female community health workers, including the work of reaching community members with vaccines [14]. Key determinants of vaccination inequity include urban slums, remote rural areas, conflict, and gender, all of which are adjudged to be responsible for more than 50% of unvaccinated and under-vaccinated children.

UNICEF, being a pro-equity agency that ensures no child is left behind, assisted several National Immunization Programmes (NIPs) to conduct gender barrier analyses for immunization. These analyses included identifying and generating an in-depth understanding of the gender-related barriers, as well as the root causes of gender and intersecting inequalities, pinpointing strategic entry points for field-level programming to drive structural transformation in vaccine access and utilization, contributing to under-vaccination, zero-dose children and missed opportunities for vaccination. The approach to conducting a gender analysis varied among countries and regions, incorporating single-country or multi-country methods, including through engaging national and international consultancy firms, reviewing the pertinent literature, collecting primary data, or employing a combination of these approaches. The gender analysis process has engaged a wide range of stakeholders, including government, parents and caregivers, adolescent mothers, UNICEF staff, frontline workers and service providers, civil society, young people, and other partners. The execution of gender analyses also differs, having regional, national, and subnational levels, as well as the types of data collection, with some having primary while others having only secondary data collection methods. The findings and recommendations from these analyses contributed to several country strategy developments including the country GAVI full portfolio planning (FPP) and adaptation of programme implementation plans, and or the national immunization strategies (NIS). This laid the groundwork for practical actions that better address gender issues, helping to boost immunization rates and reduce inequalities across countries.

Gender responsiveness is the deliberate design and implementation of policies, programmes, or interventions that recognize and address the different needs, roles, and experiences of all genders. It involves actively identifying gender inequalities, integrating gender considerations into decision-making, and taking concrete actions to reduce disparities through inclusive planning, participation, resource allocation, and monitoring outcomes to ensure equitable benefits for everyone. While gender matters profoundly for vaccine equity, there have been limited, fragmented, and non-systematic analyses of gender barriers in immunization programming across countries [10,12,15], which would help to consider interventions to address the identified barriers, as well as highlight the importance of gender-responsive programming work in immunization.

Furthermore, there has been limited evidence showcasing the correlation between gender issues beyond the potential impacts on quantitative targets, such as vaccine coverage, which often overlooks the gendered realities faced by caregivers and frontline workers, limiting the understanding of the full impact. Findings from gender analyses have opened the opportunity for countries to apply a gender lens across programmes on health and immunization. Hence, this paper adds important insights into the literature body in this area by providing a systematic analysis of gender barriers affecting immunization programming, with a focus on low- and middle-income countries (LMICs).

To bridge the evidence gap and to systematically compile and assess insights and trends from these gender analyses, this paper aims to summarize and analyze findings and recommendations from the various gender barrier analyses conducted across countries. This is meant to be a vital resource in strategic decision-making and in advancing global efforts toward equitable immunization practices.

## 2. Materials and Methods

This study employed a qualitative approach to synthesize and analyze gender barrier assessment reports from multiple countries.

### 2.1. Data Sources

From 2021, a group of countries were selected based on expressed interest and identified needs to conduct a gender analysis within their immunization or health programmes. This demand-driven approach ensured strong alignment with national priorities and was contingent on demonstrated government commitment and ownership. Each of these countries actively sought technical support from UNICEF Headquarters, underscoring their dedication to advancing gender-responsive programming and addressing equity gaps in health service delivery. All gender analyses that were conducted between 2021 and 2025 with UNICEF support were included in this synthesis. In total, 29 countries were finally included in the documents (Afghanistan, Bangladesh, Benin, Bolivia, Burundi, Egypt, Ghana, Indonesia, Iraq, Kyrgyzstan, Laos, Liberia, Malawi, Mauritania, Mongolia, Nepal, Nigeria, Pakistan, Papua New Guinea, Philippines, Rwanda, Solomon Islands, Somalia, Sri Lanka, Sudan, Syria, Tajikistan, Yemen, and Zimbabwe).

### 2.2. Data Extraction

A template developed through an iterative process by the authors of this paper was customized for this synthesis and used for data extraction. It included components such as bibliographic information and methodological aspects (including the uses of participatory approaches, the types of methods, the uses of conceptual frameworks, and the country/setting in which the analyses were conducted). The template was also piloted with five initial documents and was modified accordingly to capture all the information that was needed to conduct the data analysis.

For each gender barrier analysis, a qualitative coding approach was used to identify common gender barriers across the text. In this context, a “gender barrier” is defined as having three main characteristics:

• A clear mention of an outcome that is hindered by the existence of the barrier (e.g., immunization rates).
• A clear element that is creating a hurdle in the implementation process (i.e., the barrier itself).
• The barrier has a clear element that is connected with gender (in)equality.

This definition entails that texts related to the evaluation of programmes or interventions might not necessarily include the mention of a barrier. Similarly, recommendations from each report were extracted. Only recommendations that entailed concrete changes were considered for inclusion.

Finally, the country in which the gender analysis was conducted was classified by income level, following the World Bank country classification [16] and by its fragility, following the World Bank’s classification for the fiscal year 2025 [17].

### 2.3. Data Analysis

With the information collected from the data extraction process, a descriptive analysis with numbers and frequencies was used, while an inductive qualitative iterative coding was used to create common codes around gender barriers.

Barriers were then classified using the Global Gender Analysis Tool developed by UNICEF to assess gender-related barriers and drivers to immunization [18]. This tool is a comprehensive resource designed to help UNICEF offices, governments and partners systematically collect and analyze gender data to inform immunization efforts across diverse regions and countries. This global standard tool provides essential guidance for conducting a gender analysis that explores the root causes of gender and intersecting inequalities, identifying key entry points for field-level programming that can drive structural change in vaccine access and usage.

Using this framework, the barriers and recommendations found in this synthesis were classified according to five main socio-ecological levels:

• System level: Legal frameworks and governmental policies related to national immunization strategies.
• Health-services level: The availability, access, and quality of health services to provide immunization services, and its variation among men and women. This would include health workforce barriers (i.e., considering that the majority of them are female).
• Community level: Gender roles, cultural and religious norms within a society.
• Household level: The role of household members (e.g., parents, grandmothers, mothers-in-law, etc.) and imbalances between fathers and mothers in childhood vaccination.
• Individual level: Individual’s knowledge and education about vaccination.

## 3. Results

### 3.1. Descriptive Statistics About the Studies Included

**Figure 1:**
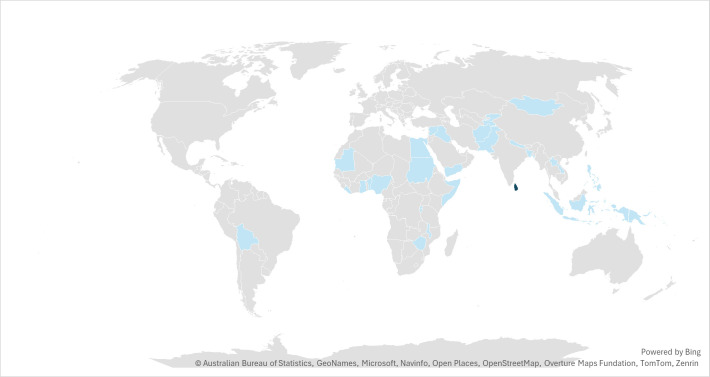
Map showing the 24 countries that are included in this evidence synthesis.

Twenty-four gender barrier analysis reports covering 29 countries were included in this synthesis (Table 1 & Figure 1), of which 24 are single country reports while 3 were regional or multi-country reports (EAPRO, MENARO, and ROSA Table 1) Figure 1.). Most analyses were conducted in lower-middle-income and low-income countries; only one was conducted in an upper-middle-income country (Indonesia). While 23 of the analyses were conducted in countries with no fragile conditions, 10 (30%) of them were conducted in a conflict or institutional and social-fragility condition.

More than 80% of the 24 documents were standalone gender analyses, rather than parts of broader studies. Four included a gender component within a maternal and child health analysis. Gender analyses commonly used a combination of methods that included key informant interviews, focus groups, desk reviews, and surveys, while they mostly employed mixed methods for data analysis. More than 70% of the gender analyses used participatory approaches, most commonly focus group discussions. None used quantitative methods alone. While some analyses did not explicitly report a specific framework used to code data and analyze results, they still organized barriers according to at least some levels of the socioecological model.

### 3.2. Summary of Barriers Identified

Across all the analyses included in this synthesis, 26 main barriers were identified and grouped according to the socio-ecological model’s five levels. Table 2, Table 3, Table 4, Table 5 and Table 6 present each barrier and specify its mechanism of action. Table 7 introduces barriers that do not inherently have a gender component but, when experienced alongside existing gender barriers, create or exacerbate gender imbalances (for example, societal beliefs against vaccination are not necessarily gender barriers, but because women are more frequently exposed to such beliefs, they are more affected by the resulting misinformation).

Nineteen gender analyses reported on at least one of the five system-level barriers (Table 2). The only demand-side barrier at the system-level identified came from Afghanistan and Indonesia, reporting that systemic discrimination against women in public spaces prevents them from accessing healthcare services, by limiting women’s mobility to access vaccination services, and significant levels of discrimination against women within healthcare settings. In terms of supply-side barriers, the lack of political will of local authorities, as well as the lack of sex-disaggregated and gender data hinders government action to develop gender-relevant policies, while banning outreach vaccination efforts affects the ability of the health system to reach the target population and increase vaccination coverage.

Twenty-six gender analyses reported on at least one of the eight health services-level barriers identified (Table 3). Six barriers affecting the demand for vaccination services were identified. Common issues arising included the discrimination and stigma against women in health facilities (mainly reported by girls and adolescents in getting their HPV vaccine), the preferences of users for the sex of a healthcare worker, and the challenges that women face in accessing healthcare and vaccination centres when healthcare workers are not trained in gender equality issues. The latter is particularly relevant, considering that it might undermine the quality-of-service delivery, eroding trust in the health system, and significantly hindering the uptake of vaccination services. Among the supply-side barriers, insecurity in commuting to work, female healthcare personnel reporting work-related challenges, as well as poorly tailored outreach activities were part of the topics raised by all analyses.

Twenty-nine gender analyses reported on at least one of the six barriers connected with the community level (Table 4). The first two barriers are connected with big societal norms that limit the ability of women to get vaccinated or to take their children to be vaccinated. These two barriers (mothers listening to multiple family members and women’s household roles) act directly to reduce women’s access to vaccination services. Gender-based violence, restricted exposure to public spaces, and societal beliefs about vaccination are also community-level barriers affecting the demand of vaccination services.

Twenty-four gender analyses reported on at least one of the three barriers associated with the household level (Table 5). All of the barriers included limit the ability of women to get vaccinated or to take their children to be vaccinated. Hence, these barriers act directly to reduce women’s access to vaccination services. As women are seen as the ones responsible for household duties and vaccinating their children, they may face economic losses, particularly if they are employed and need to take time off or deprioritize household duties when they take their children to be vaccinated. Additionally, because males are the primary household decision-makers in these settings, women need to get permission from their partners to go outside their house or also ask for males to cover transportation costs to access vaccination services. Finally, the lack of autonomy of adolescent girls inside their households limits their ability to access HPV vaccination.

Twenty-five gender analyses reported on at least one of the three gender barriers connected to the individual level (Table 6). Low levels of education among caregivers (who were mostly women) limit their access to knowledge about vaccination benefits and side effects. Additionally, multiple misconceptions were present in children and adolescents about the HPV vaccination, which reduces their willingness to get vaccinated. Finally, limited access to vaccination information is a critical gender-related barrier, as women may have less exposure to public campaigns, and their thinking could be influenced by their spouses and their families.

Finally, while several barriers reported by the gender analyses were not inherently gender-related, some of them become gender-specific when intersecting with gender dynamics (Table 7). First, some countries have reported a closure in outreach vaccination efforts. Banning of outreach vaccination efforts is commonly accompanied by a shift to allowing receiving vaccines only in certain places (e.g., mosques), which reduces access to vaccination, thereby compounding the difficulties already faced by women. This situation disproportionately affects women by reducing their access even more. Secondly, and similar to the previous point, multiple barriers that affect the access, opportunity, and quality of care would disproportionately affect women. Considering that multiple community and household-level gender barriers affect women’s access to vaccination services, having reduced hours of operations, longer waiting times and difficult physical access to healthcare facilities would significantly affect women’s access to vaccination services. Finally, as was reported in previous sections, women lack the decision-making power to seek care and vaccination services. The opinions of multiple family and community actors influence this. Hence, the misconceptions that these multiple actors might have would disproportionately affect women.

**Table 7:** Non-gender barriers that create gender barriers.

#	Barrier	How the Barrier Operates and Its Connection with Gender Barriers
1	Banning outreach vaccination efforts	Banning of outreach vaccination efforts is commonly accompanied by a shift to allowing vaccination only in certain places (e.g., mosques), which reduces the points of access to get vaccinated. This is often accompanied by restrictions for women to enter these places, which affects their access to getting vaccinated.
2	Access and opportunity of healthcare services	Low access and opportunity of healthcare services might affect those seeking care behaviour, which is directly connected to female caregivers. Then, issues such as the following arise: Hours of operation of vaccination centres for working mothers. Healthcare infrastructure ensuring close access to healthcare facilities. Efficient use of the time while the user is at the facility (e.g., not going once for registration, and then a separate time for vaccination). Considering that most caregivers are women, these factors might affect vaccination service access.
3	Quality of healthcare services provided	In cases where the quality delivered is not appropriate, this might defer female caregivers from seeking care (including vaccination). This includes inadequate infrastructure to provide privacy to female users.
Community level		
4	Societal beliefs about vaccination	Multiple misconceptions have been reported about vaccination. Considering that societal norms make women less autonomous in their decision-making (which includes vaccination), they can be more exposed to these misperceptions.

### 3.3. Gender Barriers Specific to Adolescent Girls and Fragile Contexts

Some of the barriers identified above are particularly relevant to HPV vaccination efforts and adolescent girls, as well as countries with fragile conditions. Three of the gender analyses included in this synthesis are from countries classified as conflict-affected (Afghanistan, Nigeria, and Somalia), while three others are from countries experiencing institutional and social fragility (Solomon Islands, Papua New Guinea, and Zimbabwe). Here, we describe these barriers with a specific focus on these two relevant contexts, structuring them by the same five levels that have been used before.

At the system level, the lack of political will or commitment can hinder the development of gender-awareness policies. These factors can shape HPV vaccination efforts by diverting attention from the prioritization of context-specific strategies and thereby missing critical opportunities to centre adolescent girls in vaccination policy development. At the same time, countries that are severely restricted by the fragility of their contexts or by specific restrictions imposed on women can experience an even stronger impact of the absence of gender-awareness policies in affecting their mobility to seek care. On the other hand, collecting gender data is also challenging in fragile settings, which hampers the understanding of gender dynamics in immunization.

At the health services level, some adolescent mothers also report experiencing stigma when visiting healthcare centres, while adolescent girls consistently express a preference for female healthcare providers, primarily due to safety concerns. On the other hand, security concerns intensify barriers to accessing vaccination services in conflict or fragile contexts. While geographical barriers exist in many countries, the threat of violence or conflict adds an additional layer of difficulty, as people may feel unsafe travelling to health centres.

At the community level, social norms play a critical role in creating barriers to HPV vaccination, often due to misinformation about HPV vaccination, wrongly associating it with infertility or promiscuity among girls who receive the vaccine, which can be particularly relevant in countries with strong religious norms. At the household level, a major barrier to HPV vaccination is the lack of autonomy among adolescent girls, with family views strongly influencing vaccination decisions.

Finally, at the individual level, two main barriers were identified for HPV vaccination efforts. First, limited knowledge or misinformation about HPV vaccination among adolescent girls and their families reduces willingness to vaccinate. Second, because HPV vaccination programmes in most countries do not target boys, boys may also be influenced by misconceptions, which can contribute to stigma towards vaccinated girls. This is further compounded by a lack of reproductive health education among boys. In conflict-affected settings, access to health literacy—which is essential for countering misinformation—can be significantly hindered, which makes the population even more vulnerable to misinformation and myths, as health literacy initiatives often struggle to reach them.

### 3.4. Summary of Recommendations Identified

Across the gender barrier analyses, four groups of recommendations were identified in reviewing all documents. First, institutionalizing gender in immunization policy to improve equity and accountability by implementing gender-responsive strategies and building better gender data infrastructure. This entails building capacity and infrastructure in the government and the public sector to provide a gender-responsive lens to policymaking, as well as better collecting and analyzing data that would allow decision-makers to have gender-related evidence to make decisions. Secondly, designing services for inclusion and access by designing outreach activities and empowering women healthcare workers. This includes the adaption of service provision to incorporate outreach activities to get vaccination services to populations with no access to health facilities, as well as addressing security concerns that women healthcare workers face at work. Third, engaging communities to shift social norms by engaging with local organizations and implementing programmes that promote positive masculinity. This entails partnering with trusted community leaders and women-led civil society groups in the planning and oversight of vaccination efforts, as well as promoting positive masculinity through community programmes that target males and boys to promote equal care for girls and boys. Finally, empowering caregivers and adolescents to improve their access and autonomy as well as fighting misinformation about vaccines. This includes using multi-platform health messaging to reach adolescents to improve their health literacy and autonomy, as well as fighting misinformation through multiple strategies, including training youth leaders and having safe, inclusive spaces where caregivers can ask questions and engage with health providers.

Table 8 describes each one of them, showing the specific actions that are included in each one of them. The gender analyses recommended institutionalizing gender in immunization policy by implementing gender responsive strategies, creating better gender data, and conducting research that can improve equity and accountability. This first group of recommendations targets system-level barriers.

The second group of recommendations aims to address health-service barriers by better designing services for inclusion and access. This group includes the reduction in immunization-access barriers with the uses of outreach activities, as well as better alignment of service hours with caregiving schedules, as well as empowering women healthcare workers to improve their gender-focused training and improving their safety in workplaces.

The third group of recommendations aims to engage communities in shifting social norms, which targets community-and household-level barriers. This recommendation includes working with local and religious organizations to strengthen gender-related efforts, as well as the implementation of programmes that promote positive masculinity across communities.

Finally, the fourth group of recommendations promotes the empowerment of caregivers and adolescents to improve health literacy among adolescents, and fighting misinformation across populations, in order to address individual-level barriers.

## 4. Discussion

### 4.1. Principal Findings and Findings in Relation to the Existing Literature

Gender-related barriers are a key piece in planning immunization services, impacting the ability of the population to access vaccination programmes and services. These barriers are deeply embedded across different layers of the health system and societies, influencing decision-making, care-seeking behaviour, and the design and delivery of health services. Recognizing and addressing these barriers is essential to achieving equitable immunization outcomes and meeting global immunization targets.

Following the socio-ecological model, this synthesis consolidated gender barriers at five key levels. At the system level, this synthesis included topics such as the limited integration of gender considerations in national immunization strategies. At the health-services level, gender analyses presented issues such as the lack of gender-responsive service delivery, limited female health workforce representation in decision-making roles, and inadequate training on gender responsiveness. At the community level, restrictive gender norms and the underrepresentation of women in community engagement platforms emerged as significant obstacles. At the household level, women’s restricted autonomy and limited decision-making power, especially concerning the mobility and health of their children, were central challenges. Finally, at the individual level, barriers include low levels of health and vaccine literacy among women and girls, and their constrained access to health information.

This is an updated report that synthesizes gender analyses to provide a consolidated summary of barriers that hinder vaccination efforts and recommendations on how to overcome them. This is the first synthesis that uses country-owned documents supported by UNICEF to produce a comprehensive status of gender barriers across multiple countries. These findings are in line with prior evidence indicating that social determinants and gender norms play a critical role in vaccination uptake. For example, previous systematic reviews have shown how maternal education, female mobility, and intra-household decision-making power are closely associated with immunization coverage among children [13]. Moreover, the existing literature emphasizes how gender-based discrimination, stigma, and structural exclusion affect both vaccine demand and service delivery [8,19]. Additionally, an overview of a systematic review exploring barriers to childhood vaccination found social and family influence as one of the groups of insights that were critical to acceptance to vaccination [20]. However, most existing syntheses have focused on reviewing peer-reviewed articles, which makes this synthesis the first effort to summarize evidence from gender reports produced in multiple countries. This UNICEF gender analysis synthesis provides a unique and valuable perspective on gender-related barriers to immunization by combining breadth, depth, and operational relevance. Drawing on 33 gender analyses across 29 countries, it identifies multi-level barriers from systemic gaps like weak sex-disaggregated data, to community norms restricting women’s mobility, household-level decision-making limitations, and individual-level challenges such as digital exclusion. Methodologically, it complements academic reviews systematically quantifying gender barriers and country-level case studies [10], by offering cross-country lessons grounded in programmatic experience. Compared with guidance documents [21], the synthesis is empirically rooted, actionable, and context-sensitive, highlighting strategies to empower female health workers, engage communities, and strengthen health systems. Together, these bodies of work provide a comprehensive understanding of gender barriers, illustrating both patterns and practical solutions for achieving equitable immunization.

### 4.2. Strengths and Limitations

This study has important strengths. First, it represents the first synthesis of gender barriers to immunization based on data from UNICEF-supported gender analyses, which covers 29 countries across multiple income levels and geographic regions. Second, the use of the socio-ecological framework enabled a refined analysis and classification of barriers across different levels, enabling a strong set of barriers that are relevant for policies and programmes. Finally, the identification of barriers that are not necessarily gender-related, but that might have a gender component in specific contexts is a key component of a comprehensive gender analysis, as it might bring additional issues that could also disproportionately affect women than men.

This study also presents some limitations. First, the methodology used in each gender analysis varied substantially in some cases, with important differences in the analysis depth, scope (stand-alone gender analysis and gender analysis embedded within broader maternal and child health assessments), and type of data sources. Additionally, not all analyses used explicit or consistent conceptual frameworks, which also limited the standardization of thematic coding. Second, this study did not conduct a quality appraisal of the original analyses, which could have been sensitive to the weight that certain findings could have on the final synthesis. Thirdly, the sampling of countries was mainly conducted by a demand-driven approach, which could exclude countries that have not necessarily shown an interest in conducting a gender analysis, but that could have critical gaps in vaccination efforts. Third, while we disclosed the names of the countries that were included in this synthesis, specific examples that would provide details on how each barrier might act were limited because these are government documents that are owned by the countries, and, hence, being singled-out specifically in some of the barriers might be sensitive for some of them. Lastly, this synthesis did not include non-UNICEF-led/supported or academic studies, which may have offered additional perspectives beyond the projects implemented by this international organization.

### 4.3. Implications for Policy and Practice

The findings of this paper have critical implications for policy and practice. First, they reinforce the need to mainstream gender considerations across all components of national immunization strategies, including policy formulation, development, and implementation, particularly in the push to reach zero-dose children with vaccines and vaccination across the life course, including expanding HPV vaccination coverage. Governments and partners should prioritize investments in sex-disaggregated and gender data where appropriate, training for health workers on gender responsiveness, and outreach strategies that are tailored to the specific needs of women, adolescents, and marginalized groups. Some studies have documented learnings from pro-equity strategies implemented by some countries, which include gendered immunization interventions for use by stakeholders to address the identified barriers [22]. Second, the synthesis also underscores the importance of engaging broader societal members, such as male caregivers, religious leaders, and community leaders, in behaviour change and demand-generation efforts. Programmes should ensure that gender norms are not only acknowledged but actively addressed through transformative approaches—such as positive masculinity campaigns, adolescent empowerment initiatives, and the inclusion of women in immunization planning and governance structures. Third, governments should increase the participation of women in high-level decision-making positions, particularly in prioritizing issues that are critical for women and girls. Fourth, the gender barriers and recommendations identified here could also be translated to healthcare programmes beyond vaccination efforts, to integrally incorporate more gender-responsive policies. Finally, governments and international agencies should be encouraged to have the results of gender analyses feed into programming and funding decisions to better contextualize gender issues that affect immunization, specifically for LMICs and fragile-state countries.

While this synthesis provides important insights to design future actions to improve vaccination efforts, interventions must be carefully tailored to the unique social, cultural, and structural realities of each context. Without this nuanced understanding, efforts risk overlooking critical factors that shape gender experiences and barriers, ultimately limiting the effectiveness of proposed solutions.

### 4.4. Implications for Future Research

Future research should look to answer complementary questions that could build on this synthesis. First, research that could evaluate the benefits, harms, and broader impact of gender-transformative strategies in addressing these barriers and increasing vaccination coverage, particularly in low-resource and fragile settings, is needed. Second, more studies are needed that could use an implementation research lens to understand what implementation strategies might be better positioned to achieve vaccination goals at different gender barrier levels, as well as testing the acceptability and feasibility of vaccine-uptake interventions addressing gender barriers. Third, there is also a need to document innovative practices and lessons learned from countries that have successfully translated gender analyses into policy and action, which could go beyond published research. Finally, additional research exploring the implications of gender analysis would help to understand its potential uses in decision-making processes.

## 5. Conclusions

This synthesis demonstrates that gender-related barriers are an important hurdle to achieving equitable immunization coverage. Using gender analyses from 29 countries, the study highlights how systemic, health service, community, household, and individual-level barriers restrict women’s and adolescents’ access and supply to vaccination services. The barriers identified were also matched with recommendations to mainstream gender considerations into immunization policies, strengthening sex-disaggregated and gender data systems, and implementing tailored outreach strategies that address the realities of caregivers and adolescents. Importantly, engaging communities, empowering female health workers, and promoting gender-transformative approaches can accelerate progress toward zero-dose reduction, HPV vaccination scale-up, and equitable access to routine immunizations.

## Figures and Tables

**Table 1:** Descriptive statistics of the gender analyses included (n = 24).

		N	%
Country			
	Afghanistan	1	3%
	Bangladesh	1	3%
	Benin	1	3%
	Bolivia	1	3%
	Burundi	1	3%
	Egypt	1	3%
	Ghana	1	3%
	Indonesia	1	3%
	Iraq	1	3%
	Kyrgyzstan	1	3%
	Laos	1	3%
	Liberia	1	3%
	Malawi	1	3%
	Mauritania	1	3%
	Mongolia	1	3%
	Nepal	1	3%
	Nigeria	1	3%
	Pakistan	1	3%
	Papua New Guinea	1	3%
	Philippines	1	3%
	Rwanda	1	3%
	Solomon Islands	1	3%
	Somalia	1	3%
	Sri Lanka	2	7%
	Sudan	1	3%
	Syria	1	3%
	Tajikistan	1	3%
	Yemen	1	3%
	Zimbabwe	1	3%
Income group			
	Upper-middle income	1	3%
	Lower-middle income	17	59%
	Low income	11	38%
Methodological aspects			
	Stand-alone gender barrier analysis	20	83%
	Analysis including gender issues and other topics	4	17%
	Use of a participatory approach	19	79%
Used an existing tool to conduct gender analysis			
	Yes	16	52%
	Did not report any framework	17	48%
Fragility of the country			
	Conflict	7	24%
	Institutional and social fragility	3	10%
	No fragile condition	23	66%
Data collection methods used			
	Desk review	23	70%
	Key informant interviews	28	85%
	Focus groups	23	70%
	Surveys	24	73%
Data analysis methods			
	Quantitative	0	0%
	Qualitative	6	18%
	Mixed-methods	27	82%
Types of insights included beside barriers			
	Recommendations	27	n/a
	Lessons learned	8	n/a
Contributing to different barrier levels *			
	System level	27	n/a
	Health-services level	27	n/a
	Community level	31	n/a
	Household level	27	n/a
	Individual level	27	n/a

* These barriers might have also contributed to the non-gender barriers that create gender barriers that are described in Table 7. N is the number of gender analysis reports in each criteria of the table rows.

**Table 2:** System-level barriers identified across the gender analyses.

#	Barrier	How the Barrier Operates to Hinder Vaccination Progress
Barriers affecting the demand of vaccination services		
1	Systemic discrimination against women in public places	The discrimination faced by women in public places prevent women from using public places, which affects their willingness to access healthcare services, including vaccination.
Barriers affecting the supply of vaccination services		
2	Political willingness to conduct actions to address gender barriers to improve vaccination (including gender responsiveness)	The little awareness and low political commitment in gender topics have in some countries produces two effects. First, they hinder the development of policies that could address gender barriers to improve vaccination. Simultaneously, they make gender-based programmes to be deprioritized from government agendas, which is reflected in having insufficient funds, which normally affect the health workforce salaries and outreach activities.
3	Lack of sex-disaggregated and gender data	Lack of sex-disaggregated and gender data that could allow gender analyses prevents governments to understand gender barriers that could affect vaccination roll-out campaigns.
4	Banning outreach vaccination efforts	See Table 7.
5	Participation of women in decision-making instances	Low participation of women in political life and service provision affects the quality of care that they ultimately receive, which includes vaccination.

**Table 3:** Health services-level barriers identified across the gender analyses.

#	Barrier	How the Barrier Operates
Barriers affecting the demand of vaccination services		
1	Discriminatory gender norms within health service providers	Women often feel discriminated and sometimes they face gender violence in health facilities, which also reduces their willingness to access vaccination services.
2	Availability of female trained workforce	Preferences of different countries for a specific gender in the healthcare worker could affect individuals seeking care behaviour. In some countries, a preference for female healthcare workers when receiving vaccination has been reported.
3	Access (and opportunity) to healthcare services	See Table 7.
4	Quality of healthcare services provided	See Table 7.
5	Preferences of people for male–female caregivers	Preferences across different people for the gender of the healthcare workers might reduce the willingness of the population to access vaccination services.
6	Lack of training in gender equality by healthcare workers	The direct outcome of this barrier is the reduction in the mass of healthcare workers that know about gender equality, but ultimately this affects the experience of users, which could end up not demanding vaccination services. This has often been seen in cases that discourage men to accompany spouses, as they might get ridiculed at the healthcare centre.
Barriers affecting the supply of vaccination services		
7	Security concerns, working conditions, and other female healthcare worker topics	Most of healthcare workers are female, and in some countries they have reported: Feeling insecure in their commute when going to work, especially when they are engaged in outreach activities. Feeling uncomfortable in providing care to male patients.
8	Untailored strategies to reach target populations	There is a lack of outreach policies that target girls and adolescents to increase their vaccination coverage. Additionally, the system often lacks the flexibility to respond effectively when populations relocate during the vaccination period.

**Table 4:** Community-level barriers identified across the gender analyses.

#	Barrier	How the Barrier Operates
1	Societal norms reducing women’s decision-making power regarding vaccination	In many communities, different actors (including family and community leaders) exert some influence on women’s decision to vaccinate her and her children. As a societal norm, women need to listen to multiple people. Religion also influences the access to vaccination for women in some countries, by restricting women’s access to healthcare settings.
2	Societal norms of women’s household role	For women responsible for household duties (including child’s vaccination), the time to get vaccinated needs to compete with household duties, and it might often not be prioritized.
3	Violence against women	Violence against women, manifested as early marriage, sexual violence, female genital mutilation, etc., often interrupts girls’ education, isolates them socially, and limits their engagement with health systems, reducing their access to accurate health information and services.
4	Barriers impacting women’s knowledge about vaccination	Structural social norms reduce the access of women to public places, reducing their exposure to vaccination information.
5	Preference of families for boys getting vaccinated over girls	In some cases, families would prefer boys’ wellbeing, and will provide them with access to healthcare services, which leads to fewer girls being offered vaccination services.
6	Societal belief about vaccination	See Table 7.

**Table 5:** Household-level barriers identified across the gender analyses.

#	Barrier	How the Barrier Operates
1	Female caregivers’ importance given to immunization This is a barrier that takes as a context barrier 2 at the community-level.	As women are most of the time caregivers, child immunization is seen as their responsibility. At the same time (and how it is presented in the community-level barriers), household duties are also women’s main responsibility. Hence, the importance that female caregivers provide to immunization is critical for getting their children vaccinated. However, when employed mothers suffer economic losses in taking time off to get their children vaccinated, they often deprioritize vaccination.
2	Males are the primary household decision-makers.	As males are the primary household decision-makers, women often need to perform the following: Get permission to go outside the house to get the child vaccinated. Ask for male support to incur transportation costs to access healthcare services. When male family members oppose childhood vaccination, it can directly limit women’s ability to access vaccination services for their children
3	Adolescents lack of decision-making autonomy	Adolescents often do not have the autonomy inside their household to decide whether they can get the HPV vaccine. Mainly adolescent girls often lack the autonomy with their parents—most often fathers—to making decisions about whether they receive the vaccine or not.

**Table 6:** Individual-level barriers identified across the gender analyses.

#	Barrier	How the Barrier Operates
1	Low levels of information and education about vaccination benefits, side effects and schedules among caregivers	Low level of education among caregivers (mostly women) limits their knowledge about the importance of vaccination for their children. Low knowledge of vaccination schedules and vaccination importance among caregivers, or low access of information for women.
2	Misconceptions among adolescent girls and boys	Misconceptions about potential side effects (e.g., fears of infertility or paralysis), and public exposure from boys to stigmatize girls who get the vaccine, further discouraging uptake.
3	Lack of knowledge-and exposure to misinformation among women-caregivers	Societal norms limiting women from accessing public venues might reduce their exposure to information, which limits their possibility of obtaining the right information about vaccination.
4	Misconceptions among men This is a barrier that takes as a context barrier 1 at the community-level.	Due to societal norms, men’s ideas about vaccination are more prominent, which can influence women’s decisions regarding vaccination.

**Table 8:** Synthesis of recommendations identified across the gender analyses.

#	Recommendations	Description
1	Institutionalize gender in immunization policy	Institutionalizing gender-responsive policies, data systems and inclusive planning mechanisms is key to improving equity and accountability. These recommendations include the following: 1. Gender-responsive strategies Governments should embed gender equity in national immunization strategies and budgets to include gender perspectives across government levels to help integrate gender perspective into policymaking. 2. Generating better gender data and research There is a need for reliable sex- and age-disaggregated data to track progress and inform decisions for advancing immunization initiatives and better addressing disparities.
2	Design services for inclusion and access	Designing health services to address immunization-access barriers and empowering women healthcare workers are critical areas to improve immunization. These recommendations include the following: 1. Reducing immunization-access barriers Multiple actions to improve women’s access to vaccination services have been implemented, such as mobile outreach and local vaccination points in hard-to-reach areas, or aligning service hours with women’s caregiving schedules, as well as integrating services with maternal and child health platforms. 2. Empowering women healthcare workers Safety concerns raised by female health workers can be addressed by transport support and protective protocols. On the other hand, training needs among healthcare workers need to include respectful care, adolescent-friendly communication and support for gender-based violence survivors.
3	Engage communities to shift social norms	Local communities are a key actor to consider when addressing gender community-level barriers to improve vaccination. These recommendations include the following: 1. Working with local/religious organizations to strengthen gender-related efforts Effective community engagement requires partnering with trusted male and female leaders, as well as women-led civil society groups in planning and oversight. 2. Implementing programmes to promote positive masculinity Multiple programmes targeting males and boys can be implemented, such as storytelling and peer education to challenge stigma, particularly around HPV, and to promote equal care for girls and boys.
4	Empower caregivers and adolescents	Empowering caregivers and adolescents can be critical to fighting misinformation and improving health literacy. These recommendations include the following: 1. Increasing adolescents’ agency and health literacy Using multi-platform, low-literacy health messaging as well as school-based education are key strategies, particularly to reach adolescents in improving health literacy. Governments should expand health education and review consent protocols—where legally permissible—to improve adolescent access and autonomy. 2. Fighting misinformation and increasing health literacy Fighting misinformation should be conducted with multiple strategies, including training youth leaders to share accurate vaccine information and serve as role models, as well as having safe, inclusive spaces where caregivers can ask questions and engage with health providers.

## Abbreviations

The following abbreviations are used in this manuscript:

EAPRO	East Asian and Pacific Regional Office
HPV	Human Papilloma Virus
IA2030	Global Immunization Agenda 2030
LMICs	Low-and-middle income countries
MENARO	Middle East and North Africa Regional Office
ROSA	Regional Office for South Asia
SDGs	Sustainable Development Goals
SPs	Strategic priorities
NIPs	National Immunization Programmes
